# Rapid diversification and secondary sympatry in Australo-Pacific kingfishers (Aves: Alcedinidae: *Todiramphus*)

**DOI:** 10.1098/rsos.140375

**Published:** 2015-02-04

**Authors:** Michael J. Andersen, Hannah T. Shult, Alice Cibois, Jean-Claude Thibault, Christopher E. Filardi, Robert G. Moyle

**Affiliations:** 1 Department of Ecology and Evolutionary Biology and Biodiversity Institute, University of Kansas, Lawrence, KS 66045, USA; 2 Natural History Museum of Geneva, Department of Mammalogy and Ornithology, CP 6434, CH-1211 Geneva 6, 6434, Switzerland; 3 Muséum National d'Histoire Naturelle, Département Systématique et Evolution, UMR7205, Case Postale 51, 55 Rue Buffon, 75005 Paris, France; 4 American Museum of Natural History, Center for Biodiversity and Conservation, Central Park West at 79th Street, New York, NY 10024, USA

**Keywords:** island biogeography, diversification rates, divergence time estimation, great speciators, *Todiramphus chloris*

## Abstract

*Todiramphus chloris* is the most widely distributed of the Pacific's ‘great speciators’. Its 50 subspecies constitute a species complex that is distributed over 16 000 km from the Red Sea to Polynesia. We present, to our knowledge, the first comprehensive molecular phylogeny of this enigmatic radiation of kingfishers. Ten Pacific *Todiramphus* species are embedded within the *T. chloris* complex, rendering it paraphyletic. Among these is a radiation of five species from the remote islands of Eastern Polynesian, as well as the widespread migratory taxon, *Todiramphus sanctus*. Our results offer strong support that Pacific *Todiramphus*, including *T. chloris*, underwent an extensive range expansion and diversification less than 1 Ma. Multiple instances of secondary sympatry have accumulated in this group, despite its recent origin, including on Australia and oceanic islands in Palau, Vanuatu and the Solomon Islands. Significant ecomorphological and behavioural differences exist between secondarily sympatric lineages, which suggest that pre-mating isolating mechanisms were achieved rapidly during diversification. We found evidence for complex biogeographic patterns, including a novel phylogeographic break in the eastern Solomon Islands that separates a Northern Melanesian clade from Polynesian taxa. In light of our results, we discuss systematic relationships of *Todiramphus* and propose an updated taxonomy. This paper contributes to our understanding of avian diversification and assembly on islands, and to the systematics of a classically polytypic species complex.

## 2. Introduction

Classic hypotheses about diversification of insular organisms are based on a relatively simple dynamic between colonization and extinction of allopatrically derived species [1–6]. These ideas are being challenged, however, by phylogenies that support complex diversification and colonization scenarios (e.g. [7–9]). This re-evaluation of insular diversification has revealed extensive insular radiations with high sympatric diversity and subsequent re-colonization of continental areas. Thus, community diversity on islands depends not only on the flow of colonists from continental areas, but also on the frequency of secondary sympatry within insular lineages. Furthermore, a broad spectrum of lineage ages exists in island systems. For example, recent phylogenetic study has uncovered the ubiquity of insular avian lineages exhibiting recent, allopatric diversification over large areas of the Pacific [10–12], providing a context for high potential speciation rates. Conversely, ‘mature’ insular radiations exist with extensive co-occurrence of constituent taxa and substantial ecomorphological differentiation, which often confounded traditional taxonomy [7,12–15].

The rate of attaining reproductive isolation and the build-up of sympatry (i.e. assembly) on islands is understudied in non-adaptive radiations (e.g. away from Hawaii and the Galápagos; [16–18]). A key component is the critical stage after initial geographical expansion and subsequent diversification (i.e. allopatric speciation) when diversifying lineages initiate secondary sympatry among recently diverged populations. Unfortunately, most avian radiations are not suitable for studying the process of secondary sympatry on islands. For example, mature insular radiations provide only an incomplete picture because extinction, changes in distribution and substantial anagenesis obscures early stages of lineage accumulation, whereas purely geographical radiations (e.g. [10]) have not yet begun the process; thus, they are uninformative in the study of insular species assembly and secondary sympatry. Evidence from mature continental radiations supports a scenario of substantial divergence in allopatry before lineages are able, or have the opportunity, to co-occur [19,20]; however, factors that influence rates to secondary sympatry in continental systems are numerous: complex geography, closed ecological communities, disease transmission, biotic and abiotic environmental interactions, ecological similarity of sister taxa and complex signalling environments [20–25]. Conversely, insular systems are comparatively simple and may provide the most accessible insight into the tempo and mode of attaining secondary sympatry, even though extrapolation to diverse continental systems is difficult [17].

Here, we examine the phylogeographic and temporal patterns of diversification in the *Todiramphus chloris* species complex (Aves: Alcedinidae) and its close relatives. This species complex is the most widespread of the archetypal ‘great speciators’ [26], and comprises 50 nominal subspecies spanning a distance more than 16 000 km from the Red Sea to Samoa [27–29]. The full geographical extent of the genus extends a further 3000 km east to the Marquesas Islands in Eastern Polynesia (kingfishers do not occur in Hawaii). Most nominal subspecies correspond to single-island populations that are phenotypically distinct in plumage and size, but some islands/archipelagos have multiple sympatric *Todiramphus* species, including Palau, Vanuatu, and several islands in the Solomon Islands and the Bismarck Archipelago, as well as Australia. These instances of sympatry are presumed to be secondary (i.e. after allopatric speciation). Additionally, the distribution of *Todiramphus sanctus*—the only migratory *Todiramphus*—broadly overlaps many congeners in the *T. chloris* complex. All sympatric *Todiramphus* exhibit ecological, morphological and behavioural differences, including separation by habitat preference, suggesting a high degree of reproductive isolation between each pair [27,28,30]. Previous phylogenetic work on higher level kingfisher relationships showed extremely low genetic differentiation among five *Todiramphus* species [31], but only one *T. chloris* sample was included. With regard to non-adaptive (e.g. geographical) insular radiations, the *T. chloris* complex has several notable features. The broad distribution, numerous instances of closely related, sympatric species and close relationship between migratory and sedentary species make the *T. chloris* complex an ideal lineage for examining the consequence of rapid diversification and subsequent assembly of secondarily sympatric species in an insular system.

## 3. Material and methods

### 3.1 Taxon sampling

**Figure 1.**
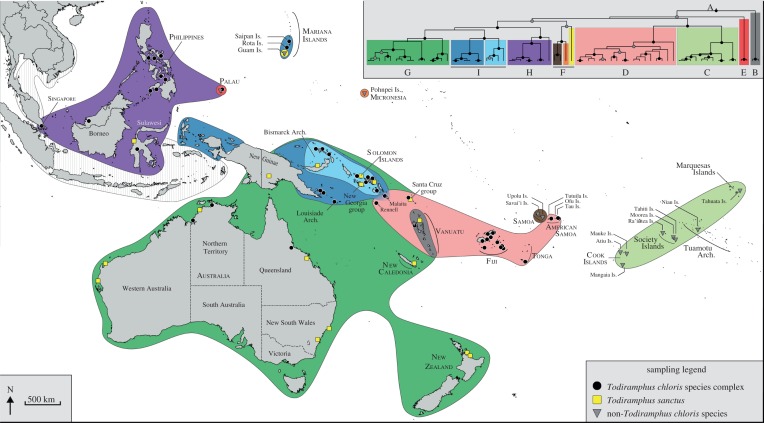
Map illustrating sampling of the *Todiramphus chloris* species complex used in this study. Circles, squares and triangles represent sampling points for *T. chloris*, *T. sanctus* and other (non-*T. chloris*) ingroup taxa, respectively. Coloured distributions correspond to the 11 major phylogenetic lineages of the *T. chloris* species complex and match the coloured clades in the inset phylogeny. The inset topology is from the BEAST tree (figure 3) with clades labelled A–G matching those from the MrBayes tree (figure 2). Points are not scaled to the number of sampled individuals per locality (the reader is referred to the electronic supplementary material, table S1 for numbers of individuals sampled).

Our taxon sampling comprised 158 individuals (electronic supplementary material, table S1; figure 1), including one *Actenoides*, two *Syma* and 155 *Todiramphus* samples. Of the 155 *Todiramphus* samples, 93 were *T. chloris* and 62 were composed of 15 additional *Todiramphus* species. We lacked only six *Todiramphus* species (*T. diops*, *T. lazuli*, *T. albonotatus*, *T. funebris*, *T. enigma* and *T. australasia*), owing to their distribution in areas where collecting fresh genetic source material is difficult. Our *T. chloris* sampling included 22 of 50 nominal subspecies [29]. Moyle [31] showed that *Todiramphus* is a clade distinct from *Halcyon* and sister to *Syma*; therefore, we used *Actenoides hombroni*, *Syma megarhyncha* and *Syma torotoro* as outgroups to root trees. Whenever possible, we sequenced multiple individuals per population (i.e. per island) to guard against errors of misidentification, mislabelling or sample contamination.

### 3.2 DNA sequencing, alignment and model selection

We extracted genomic DNA from frozen or alcohol-preserved muscle tissue, toepads of museum study skins or unvouchered blood samples (electronic supplementary material, table S1) using a non-commercial guanidine thiocyanate method [32]. For toepad extractions, we used laboratory space separate from other *Todiramphus* pre- and post-PCR products to minimize contamination risk [33]. We used unvouchered blood samples for taxa from remote islands in French Polynesia where collection of vouchered specimen material was not possible owing to small population sizes of endangered species (e.g. *Todiramphus gambieri*; electronic supplementary material, table S1; [34]). We sequenced the entire second and third subunits of mitochondrial nicotinamide adenine dinucleotide dehydrogenase (hereafter ND2 and ND3, respectively) and four nuclear gene regions: the coiled-coil domain containing protein 132 (CCDC132), the high mobility group protein B2 (HMGB2), the third intron of the Z-linked muscle-specific kinase gene (MUSK) and the fifth intron of the transforming growth factor *β*2 (TGF*β*2) following protocols described in [35]. We used the following external primers in PCR amplification and sequencing: L5215 (ND2, [36]) and H6313 (ND2, [37]), L10755 and H11151 (ND3, [38]), CDC132L and CDC132H [39], HMG2L and HMG2H [39], MUSK-I3F and MUSK-I3R [40], and TGF5 and TGF6 [41]. We modified external primers for CCDC132 and HMGB2 to better suit *Todiramphus,* and we designed internal primers to amplify 200–250 bp fragments of toepad samples (electronic supplementary material, table S2).

We assembled and aligned sequence contigs in Geneious v. 6.1 (Biomatters), constructed individual nuclear intron alignments by hand, and checked them against an automated alignment in MUSCLE [42]. We phased introns in DnaSP [43] with output threshold of 0.7 using algorithms provided by PHASE [44,45]. We identified appropriate models of sequence evolution for each of the seven partitions (electronic supplementary material, table S3) using Akaike's information criterion (AIC), as implemented in MrModelTest v. 2.3 [46].

### 3.3 Phylogenetic analysis

We performed phylogenetic reconstruction on the total concatenated data, on separate concatenated mitochondrial DNA (mtDNA) and nuclear DNA (nDNA), and separately on each locus. We performed maximum-likelihood (ML) heuristic tree searches in GARLI v. 2.0 [47] and Bayesian analysis (BA) in MrBayes v. 3.2.1 [48–50], implemented with BEAGLE [51]. We partitioned all ML and BA analyses by codon position for mtDNA and by gene for the nuclear introns. To avoid local optima in GARLI, we did 250 independent searches, each starting from a random tree. We adjusted GARLI's default parameters to terminate searches when no topological improvements were found after 100 000 generations (genthreshfortopoterm=100 000); otherwise, we used default settings. We assessed statistical support for the ML topology with 1000 non-parametric bootstrap replicates [52] and generated a 50% majority-rule consensus tree in SumTrees v. 3.3.1, part of the DendroPy v. 3.12.0 package [53]. In MrBayes, we did four independent Markov chain Monte Carlo (MCMC) runs of 25 million generations using four chains per run (nchains=4) with incremental heating of chains (temp=0.1) sampled every 2500 generations. We changed the default branch length prior to unconstrained with an exponential distribution for all partitioned analyses to avoid artificially long branches (prset applyto=(*all*) brlenspr=unconstrained:exponential(100); [54]). We assessed convergence of parameter estimates and tree splits in Tracer v. 1.5 [55] and Are We There Yet? (AWTY?; [56,57]), respectively. We assessed topology convergence between runs by the average standard deviation of split frequencies (ASDSF) and potential scale reduction factor. We discarded an appropriate number of burn-in generations based on convergence assessments of the ASDSF passing below 0.01; the remaining trees were summarized in a 50% majority-rule consensus tree.

### 3.4 Molecular dating and species delimitation

We estimated divergence time in BEAST v. 1.7.5 [58,59] implemented with BEAGLE [51]. We included two individuals per nominal subspecies for all *Todiramphus* taxa, except *T. sanctus*, for which we included only known breeding populations (e.g. Australia, New Zealand, New Caledonia, Solomon Islands, Vanuatu and the Santa Cruz group; electronic supplementary material, table S1). We linked clock and tree models, but nucleotide substitution models were unlinked. We used MrModelTest to partition the data in the same way we did our MrBayes analyses (electronic supplementary material, table S3). We used a birth–death speciation process for the tree prior. To test for clock-like evolution, we compared likelihoods of runs with a strict clock to those with a relaxed lognormal clock (UCLD). We failed to reject a strict molecular clock using a likelihood ratio test (*p*=1.0). Additionally, the coefficient of variation frequency histogram of the *ucld.std*parameter abutted against zero when viewed in Tracer, which is a symptom that the data cannot reject a strict molecular clock [60]. We ran 10 independent MCMC chains for 100 million generations and sampled every 20 000th generation. We examined burn-in and convergence diagnostics in Tracer; burn-in values were specific to each run with at least 25% of samples discarded, with some runs requiring up to 40% burn-in. Lacking fossil calibration data for this group, we relied on published rates of mtDNA sequence evolution to calibrate our divergence dating analyses. Substitution rate priors derived from ND2 substitution rates for Hawaiian honeycreepers were used (0.024 and 0.033 substitutions per site Myr^−1^; [61]). We chose ND2 because it is one of the fastest-evolving mitochondrial gene regions in birds [61] and it is used widely among avian systematists and phylogeographers. We used a lognormal prior distribution for the *clock.rate* parameter with mean=0.029 and standard deviation=0.25. Using a general substitution rate from distantly related species is not ideal (e.g. kingfishers versus honeycreepers), but we note that mtDNA substitution rates across birds cluster around this value [19,62]. Regardless, these date estimates can only be used as a rough guide to clade ages. We used separate normally distributed substitution rate calibration priors for the three ND2 codon positions, whereas the introns were scaled to the mtDNA rate priors. ND3 was omitted from BEAST analyses to simplify mitochondrial rate calibrations.

We examined species delimitation and diversification rates to objectively compare patterns of diversity in *T. chloris* to other published phylogenies of rapid geographical radiations (e.g. *Zosterops* and *Erythropitta*). We delimited species with a Bayesian implementation of the general mixed Yule-coalescent model implemented in the R package, bGMYC [63]. We used the ND2 data and followed the authors' parameter recommendations [mcmc=50 000; burn-in=40 000; thinning=100]. The GMYC model [64] is advantageous for single-locus datasets such as those generated by DNA barcodes or when the majority of phylogenetic signal occurs in the mtDNA, including rapid radiations like *Todiramphus*. We calculated diversification rates assuming a Yule process from the following formula: [ln(*N*)–ln(*N*_*o*_)]/*T*, which uses initial diversity (*N*_*o*_=2), extant diversity (*N*) and time (*T*) since origin of the crown clade [65].

## 4. Results

### 4.1 Phylogenetic relationships

Topologies inferred from multiple independent ML and BA runs were highly concordant. MCMC chain stationarity was achieved in MrBayes (i.e. the ASDSF remained less than 0.01) after 8.15 million generations. Individual nuclear gene trees were largely uninformative at this shallow scale, but both mtDNA genes (ND2 and ND3) provided good phylogenetic resolution. No conflicting topologies were strongly supported between individual gene tree analyses (results not shown).

**Figure 2.**
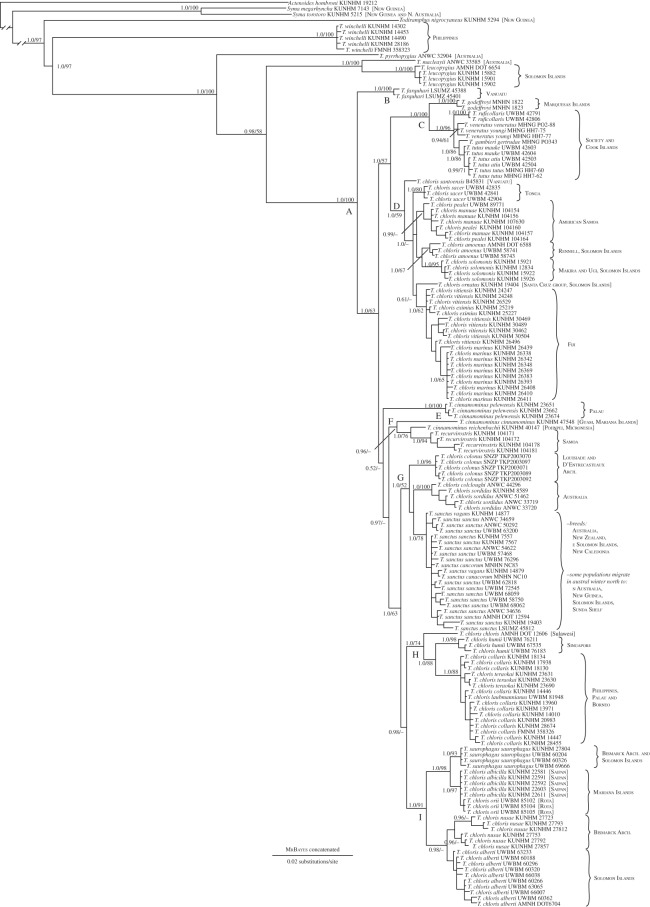
Molecular phylogeny of the *Todiramphus chloris* species complex. The tree is the Bayesian maximum consensus tree from the concatenated, partitioned analysis with full sampling (*n*=158 tips). Node support is denoted as Bayesian posterior probabilities/maximum-likelihood bootstrap support. Branch lengths of *Actenoides* and *Syma* were reduced to save space. Lettered clades (A–I) are discussed in the text.

The ingroup included all *T. chloris* samples plus 10 additional *Todiramphus* species (figure 2, clade A: posterior probability (PP) =1.0, bootstrap support (BS) =100). We defined this focal clade inclusive of *T. farquhari* because this circumscribed a suite of 11 closely related species subtended by a long internode that separated them from all other *Todiramphus* taxa. Multiple instances of sympatry exist within the focal clade, including on Australia (*n*=2 taxa, plus two outgroup taxa), Palau (*n*=2), the Solomon Islands (*n*=2, plus 1 outgroup), the Santa Cruz group (*n*=2) and Vanuatu (*n*=2; figure 2).

Clade A contained seven subclades (figure 2, clades B–I), each with PP=1.0, except clade F (PP=0.96), which includes *T. cinnamominus* from Guam and Pohnpei, and *T. recurvirostris* from Samoa. Of the 10 non-*T. chloris* species in the focal clade, clade C comprised five species endemic to Eastern Polynesia: *T. godeffroyi*, *T. ruficollaris*, *T. veneratus*, *T. gambieri* and *T. tutus*. Clade D was sister to clade C and comprised *T. chloris* lineages from Central Polynesia, inclusive of American Samoa, Tonga, Fiji, Vanuatu and the eastern Solomon Islands including Makira, Ugi and Rennell Islands, and the Santa Cruz group.

The placement of clades E and F was equivocal. The three subspecies of *T. cinnamominus* were split between these clades, rendering the species paraphyletic. The Palau endemic, *T. c. pelewensis*, was the sole member of clade E, whereas *T. c. cinnamominus* and *T. c. reichenbachii*, island endemics of Guam and Pohnpei, respectively, were sequentially sister to *T. recurvirostris*, itself an endemic of American Samoa. Clade G comprised *T. chloris* lineages from Australia and Papua New Guinea plus *T. sanctus*, which was embedded inside this clade. Clade H comprised three genetically distinct lineages: nominal *T. c. chloris* from Sulawesi, *T. c. humii* from Singapore, and a clade that comprised multiple subspecies from Borneo to the Philippines and Palau. Finally, clade I included lineages from such geographically disparate regions as Melanesia and the Mariana Islands. *Todiramphus saurophagus* was sister to *T. c. albicilla*+*T. c. orii* from Saipan and Rota, Mariana Islands. The other half of clade I included *T. c. nusae* and *T. c. alberti* of the Bismarck Archipelago and Solomon Islands, respectively, to the exclusion of the eastern Solomon Islands (Makira, Ugi and Rennell; clade D).

### 4.2 Divergence times, diversification rates and species limits

**Figure 3.**
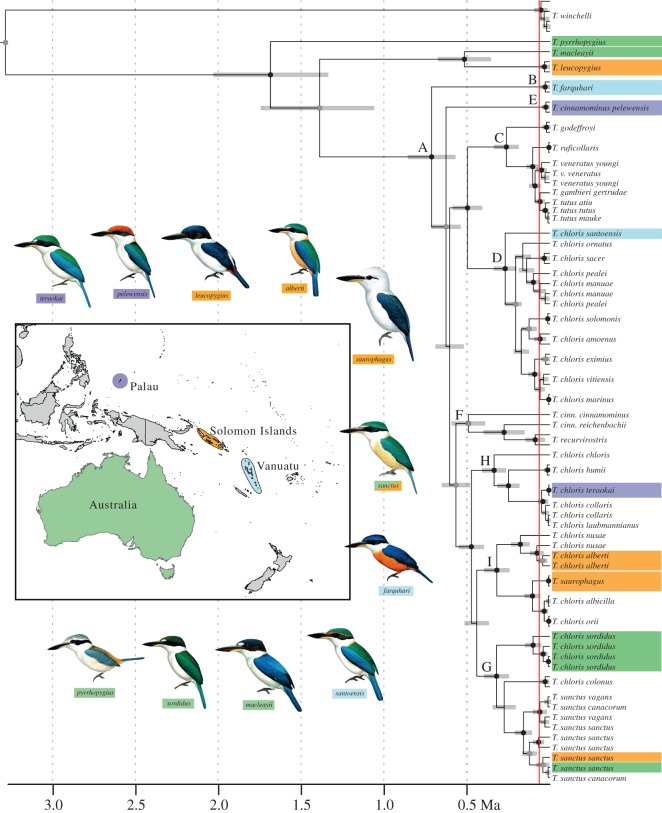
Time-calibrated maximum clade credibility tree with 95% highest posterior density bars from the BEAST analysis. Node support is given as Bayesian posterior probability (PP): black circles at nodes denote PP=1.0, grey circles denote 0.95≤PP≤0.99. Unlabelled nodes denote PP<0.95. The red vertical line denotes the bGMYC species delimitation estimate (i.e. the bGMYC analysis identified as species all clades to the right of the line). Sympatric lineages are identified by colour-coded labels that correspond to their respective distributions on the map. Note that *T. sanctus* is distributed across two coloured areas (green Australia and orange Solomon Islands). *Actenoides hombroni*, *Syma* and *Todiramphus nigrocyaneus* were removed from the base of the tree to save space. Lettered clades (A–I) are discussed in the text and correspond to the same clades in figure 2. Illustrations of the sampled lineages from Palau (*T. c. teraokai*) and Vanuatu (*T. c. santoensis*) were not available, so representative taxa from their respective clades were used (*T. c. chloris* and *T. c. juliae*, respectively). Illustrations courtesy of the *Handbook of the Birds of the World*, Lynx Edicions.

 *Todiramphus* diversified rapidly and recently. The ND2 sequence divergence within the focal clade (clade A) was 2.2% (median ND2 uncorrected P distance between *T. farquhari* and all remaining clade A taxa). The maximum pairwise divergence (3.4%) occurred between the Southeast Asian clade, including nominate *T. c. chloris* (clade H) and the eastern Polynesian clade (clade C). We used two rates of ND2 sequence divergence derived from the 95% CI range from Hawaiian honeycreeper mitogenomes (0.024 and 0.033 substitutions per site Myr^−1^; [61]) to calibrate the clock prior in our BEAST analysis. The faster rate (3.3%) results in a younger age estimate, whereas the slower rate results in an older estimate. These calibrations place the start of diversification of clade A in the mid-Pleistocene, approximately 0.57–0.85 Myr ago (mean 0.71 Ma; figure 3). We caution against strict interpretation of these values because divergence time estimation based on a molecular clock has numerous shortcomings, especially when based on single-gene calibrations from distantly related species, as well as in the absence of fossil or island-age calibrations.

Threshold species delimitation with bGMYC suggested that current species diversity is vastly underestimated in *Todiramphus*. Current taxonomic authorities [29] recognize 11 biological species that are nested within our clade A. The bGMYC estimate, based on ND2 data only, found strong support for 26 species within clade A plus seven species outside it (i.e. outgroup taxa; figure 3). This estimate of 26 ingroup species probably is conservative because we lacked 28 of the 50 nominal subspecies of *T. chloris*. We calculated two pairs of diversification rates based on estimates of species diversity in clade A: the more conservative 11 ‘bio-species’ (e.g. following current taxonomy; [29]) and our more liberal bGMYC estimate of 26 ingroup species. For each ingroup species scenario (11 and 26 species, respectively), we calculated diversification rates based on the range of crown clade ages derived from the BEAST divergence time estimation (0.57–0.85 Myr ago). Thus, our conservative estimate (*n*=11 ingroup species) yields a diversification rate of 2.01–2.99 sp Myr^−1^, whereas our bGMYC-based estimate (*n*=26 ingroup species) is 3.02–4.49 sp Myr^−1^, which surpasses the fastest speciation rates yet reported in birds [66]. If we achieved complete taxon sampling of all 50 *T. chloris* nominal subspecies, our diversification rate estimate probably would be higher.

## 5. Discussion

### 5.1 Timing and rates of diversification

Phylogenetic results indicate that characterization of *T. chloris* as a ‘great speciator’ [26] was not quite accurate, because *T. chloris* is not a natural group. Indeed, the reality is even more striking; 10 species were found to be embedded within or minimally divergent from *T. chloris*, rendering it paraphyletic. Unbeknownst to Diamond *et al*. [26] in their description of the paradox of the great speciators, rapid geographical diversification of the *T. chloris* complex was accompanied by several instances of secondary sympatry involving morphologically disparate taxa (figure 3), which obscured their evolutionary relationships. Phylogenetic reconstruction and molecular dating estimates revealed that the *T. chloris* complex is extremely young and reached its geographical distribution quite rapidly. The divergence between *T. farquhari* and the rest of the ingroup was only 2.2% (ND2 uncorrected P), which yielded a crown clade divergence time estimate for the complex between 0.57 and 0.85 Ma. This time frame in the mid-Pleistocene is more recent than the diversification of the red-bellied pitta *Erythropitta erythrogaster* throughout the Philippines, Wallacea and New Guinea (approx. 1.8 Ma; [10]). However, we caution against drawing specific conclusions based on these time estimates because of myriad shortcomings of molecular clock calibrations for divergence time estimation [67–69]. Nevertheless, our estimates of divergence time and species-level diversity (i.e. unique evolutionary lineages) produced high diversification rate estimates compared with other birds [70]. Overall, we interpret the striking pattern of shallow internodes at the base and relatively shallow divergences between ingroup taxa as support for a scenario in which *Todiramphus* achieved its full geographical distribution—from French Polynesia to the Sunda Shelf (and possibly the Red Sea, although those populations were not sampled)—rapidly and recently. Similar patterns have been noted in other Pacific bird lineages, including *Acrocephalus* reed-warblers [71], *Alopecoenas* doves [72,73], *Ceyx* kingfishers [11], *Erythropitta* pittas [10], *Pachycephala* whistlers [35,74] and *Zosterops* white-eyes [66]. However, not all Pacific bird lineages fit this pattern of rapid and widespread diversification; monarch flycatchers [7] and *Ptilinopus* fruit-doves [75] are two examples of widespread, ‘mature’ lineages that have been diversifying throughout the Pacific for much longer.

### 5.2 Secondary sympatry, shifting dispersal ability and migration

Reduction in dispersal ability or propensity after geographical expansion is a leading hypothesis for diversification of rapid geographical radiations in island settings [6,26,76]. Although rapid reduction of dispersal ability would allow for differentiation among island populations, it would seemingly prevent secondary colonization that is required to achieve sympatry. This key evolutionary juncture is where the paradox of the great speciators [26] and the taxon cycles hypothesis [6] intersect: together, these hypotheses allow for differentiation and build-up of secondary sympatry with repeated colonization. Among insular avian radiations, a clear dichotomy exists between lineages that underwent expansive geographical differentiation but rarely or never attained secondary sympatry [10,11,35,74], and those that display both broad geographical diversification as well as build-up of sympatric diversity [7,13,15,75]. This can be seen in the *Ceyx lepidus* species complex (Aves: Alcedinidae), which has geographical replacement populations across approximately 5000 km of the southwest Pacific, but has only attained sympatry with close relatives in portions of the Philippines [11]. Like *Todiramphus,* the phylogeny of *C. lepidus* has shallow internodes at the base with long branches subtending extant island populations. This pattern is consistent with rapid geographical expansion followed by reduction in dispersal ability across all of *C. lepidus*. Based on our molecular dates, *C. lepidus* is about twice as old as the entire *T. chloris* radiation. Clade age can affect interpretation of diversification rate [77,78], but it appears that *C. lepidus* is a lineage whose diversification slowed after an initial stage of rapid geographical expansion.

Reduction in dispersal propensity, however, need not proceed uniformly across a clade. Indeed, rails, *Ptilinopus* fruit-doves and *Zosterops* white-eyes, show marked differences in dispersal ability among closely related lineages across the Pacific [66,75,79–81]. Importantly, all three groups also have substantial secondary sympatry among species (rails did prior to widespread extinction), which coincides with dispersive taxa. Wilson [82] noted the possibility of this uneven change in dispersal ability within a diversifying lineage in the context of cyclic expansion and contraction phases in diversification. The layering of *Todiramphus* taxa resulting from such cycles is best illustrated in the Solomon Islands. Four *Todiramphus* species breed on the larger islands and are clearly differentiated by age, habitat and inferred dispersal propensity (figure 3).

In the context of Diamond's [26] and Wilson's [6] views on the influence of variable dispersal abilities on diversification patterns, the *T.* *chloris* complex contains multiple instances of secondary sympatry that juxtapose taxa with markedly different dispersal histories. The incidence of secondary sympatry across the Pacific distribution of the *T. chloris* group is remarkably high given the recency of the radiation. In every case, the sympatric lineages diverged substantially in terms of phenotype, morphology, ecology, dispersal ability/propensity and/or behaviour. For example, Palau holds two *Todiramphus* species: *T. cinnamominus pelewensis* and *T. chloris teraokai.* These taxa have diverged morphologically and in habitat preference, such that *T. c. pelewensis* is *ca*. 50% smaller in body mass and inhabits forest interior, whereas *T. chloris teraokai* is larger and prefers coconut groves and beaches [30,83]. The species differ in plumage as well: *T. c. pelewensis* has an orange crown, whereas *T. chloris teraokai* has a blue-green crown typical of many *T. chloris* forms. A difference in dispersal history can be inferred from distributions and genetic structure of the two taxa: *T. c. pelewensis* is restricted to the Palau Archipelago and a relatively large genetic divergence separates it from its nearest relative. By contrast, *T. chloris teraokai* is embedded in a relatively undifferentiated clade that also spans the Philippine archipelago and Borneo. It appears that Palau was first colonized by *T. cinnamominus*, with *T. chloris* arriving quite recently (figure 3). This nested pattern of old and young lineages within an archipelago was also noted recently in *Ptilinopus* fruit-doves from Fiji and Tonga [75].

The beach kingfisher, *Todiramphus saurophagus*, which is broadly sympatric with the *T. chloris* clade from the Bismarck Archipelago and Solomon Islands, displays a similar pattern. *Todiramphus saurophagus* is the largest species in the genus; it is twice the size of the sympatric *T. chloris* forms, and it differs phenotypically from most other *Todiramphus* in having a completely white head (save a blue post-ocular stripe). It inhabits beaches, coastal forest, reefs, islets and occasionally mangroves, but never ventures far from the coast. Throughout its distribution from the northern Moluccas to the Solomon Islands, it is sympatric with one to two species of *Todiramphus*, including representative *T. chloris* forms. For example, *T. chloris alberti* and *T. chloris nusae* occur in the Solomon Islands and Bismarck Archipelago, respectively, where they inhabit secondary forest and open areas away from the coast. Notably, *T. saurophagus* and both *T. chloris* subspecies are in the same subclade of the *T. chloris* phylogeny and diverged from one another quite recently, perhaps 0.5 Ma (figure 3).

The most complex scenario of secondary sympatry in *Todiramphus* occurs in clade G (figure 2). This clade comprises all *T. chloris* from Australia and New Guinea, which are split in two lineages: (i) an endemic to the Milne Bay Province islands of southeast Papua New Guinea, *T. c. colonus*; and (ii) the Australian clade, *T. c. sordidus*+*T. c. colcloughi*. These allopatric lineages occur in different habitats: forest edge on small islands in the D'Entrecasteaux and Louisiade Archipelagos (*T. c. colonus*) and mangrove forest and coastal estuaries of northern and eastern Australia (*T. c. sordidus*+*T. c. colcloughi*). *Todiramphus sanctus* is the third lineage in clade G. This species is widespread and some populations are highly migratory. Its breeding range spans Australia, New Zealand, New Caledonia and parts of the Solomon Islands. Many populations migrate north in the austral winter to the Sunda Shelf, New Guinea and Northern Melanesia. We sampled three of the five nominal subspecies [29], including two from previously unknown localities (Nendo Island, Santa Cruz group and Espiritu Santo, Vanuatu), and despite the geographical complexity of this species' distribution, there was no genetic substructure within *T. sanctus*; individuals from migratory and sedentary populations across their broad distribution are intermixed in the clade.

Sympatric forms of *T. chloris* and *T. sanctus* differ ecomorphologically and behaviourally. *Todiramphus sanctus* is smaller than any sympatric *T. chloris* throughout its range. Behaviourally, the migratory nature of *T. sanctus* is novel in *Todiramphus* kingfishers. This behaviour is particularly relevant in light of the ‘great speciators’ paradox [26]. The paradox poses the question: why are some species geographically widespread, implying high dispersal ability, but at the same time well-differentiated across even narrow water gaps, implying low dispersal ability? Diamond *et al*. [26] suggested that some of the ‘great speciators’ underwent colonization cycles in which they had past phases of higher immigration rates and dispersal abilities followed by a loss of dispersal ability with subsequent differentiation on newfound islands. They count *Todiramphus* [*Halcyon*] *chloris* among the several lineages as evidence for this idea. That the migratory *T. sanctus* is so closely related to *T. chloris*—especially given its placement deeply embedded in the phylogeny—emphasizes the potential role of shifts in dispersal ability as a driver of diversification. It is possible that the migratory nature of *T. sanctus* is an evolutionary vestige of the ancestral *Todiramphus* lineage still exhibiting the colonization phase of Diamond *et al*. [26]. If so, *T. sanctus* offers intriguing evidence in support of this component of the paradox.

Rapid reduction of dispersal ability in island birds has been suspected [66,84,85], and evidence suggests that morphological change is not necessary for such a shift in dispersal ability; it can be entirely behavioural [86]. It has also been shown that birds can acquire migratory ability quickly in response to selective pressure [87,88], and this trait is thought to be evolutionarily labile [89]. A prevailing paradigm is that extant migratory species evolved from sedentary tropical ancestors [90], however, recent evidence in emberizoid passerines suggests otherwise [91,92]. Loss of migration may be as common as gains and extant sedentary tropical radiations (e.g. some *Geothlypis* and a clade containing *Myiothlypis*, *Basileuterus* and *Myioborus*) represent at least two losses of latitudinal migration with possible colonization of the tropics from the temperate region [91].

## 6. Conclusion

Early biogeographers such as Darwin, Wallace and Darlington appreciated that lineages can diversify across vast insular systems. Subsequent observation led to description of similar patterns across many of these radiations and formulation of hypotheses to explain them (e.g. ‘Taxon Cycles’ and ‘Great Speciators’). We showed that the *T. chloris* group exhibits three characteristics of particular interest in discussions of how diversity accumulates on islands. First, the group diversified rapidly concomitant with a geographical expansion covering approximately 16 000 km of longitude. This diversification rate is among the most rapid known in birds [66,70]. Second, within the short time frame of diversification, secondary sympatry has been achieved multiple times. Although it is unmeasured in many groups, a broad survey of times to secondary sympatry in New World birds [19] reveals that *T. chloris* is exceptional in its short time to secondary sympatry. Third, extreme disparity in dispersal ability has evolved within the group—migratory *T. sanctus* is embedded within the sedentary *T. chloris* complex. Together, these aspects support a hypothesis that rapid and uneven shifts in dispersal propensity across clades have been prominent in moulding the evolution of insular biotas.

## Supplementary Material

Appendix: Biogeography and species limits; Tables 1-3.

## Data accessibility

All DNA sequences generated in this study are deposited in GenBank under accession numbers (KP291162–KP292029).
