# Supplementary material for: Rapid diversification and secondary sympatry in Australo-Pacific kingfishers (Aves: Alcedinidae: Todiramphus)
Source: R Soc Open Sci. 2015 Feb 4;2(2):140375. doi: 10.1098/rsos.140375 (PMC4448819; doi:10.1098/rsos.140375)
Supplement: Appendix: Biogeography and species limits; Tables 1-3. [file rsos140375supp1.docx]

**Electronic Supplementary Material (ESM)**

**Title**

Rapid diversification and secondary sympatry in Australo-Pacific kingfishers (Aves: Alcedinidae: *Todiramphus*).

**Authors**

Michael J. Andersen, Hannah T. Shult, Alice Cibois, Jean-Claude Thibault, Christopher E. Filardi, Robert G. Moyle

**Appendix**

(a) Biogeography and colonization of the eastern Pacific

The rapid and widespread nature of *Todiramphus* diversification across the Pacific precludes a simple stepping-stone model of colonization. Furthermore, extinction from natural or anthropogenic causes could mask underlying patterns, especially in Eastern Polynesia where small populations persist on remote islands [1]. Nevertheless, current distributions and a robust phylogeny revealed a major biogeographic break in the Solomon Islands that separates clades C and D from the rest of the ingroup. This biogeographic break implies early colonization of Polynesia, with subsequent diversification in two broadly distributed radiations from the eastern Solomon Islands to Eastern Polynesia. Clade C comprised a radiation of five species endemic to some of the most remote islands in the world. Interestingly, *T. godeffroyi,* an endemic of the Marquesas Islands—the most remote archipelago within the distribution of *Todiramphus*—is sister to the other four species in the clade: *T. ruficollaris*, *T. veneratus*, *T. gambieri*, and *T. tutus*. Each species is monophyletic, but relationships among them are equivocal. The taxonomic history of this group is muddled, likely owing to the great reluctance of BSC-influenced taxonomists to delimit allopatric insular taxa—despite the existence of fixed morphological and behavioral differences—as species. (To their credit, such an endeavor is seemingly easier today with evidence from molecular phylogenetics.) For example, *T. ruficollaris* has been treated by various authors as a nominal subspecies of *T. sanctus* or *T. tutus*, or as a full species-level taxon [2-4]. Our results show it is phylogenetically unrelated to *T. sanctus* (Fig. 2). Instead, it is part of a geographically cohesive radiation in the Cook and Society Islands that comprises *T. tutus* and *T. veneratus*, plus *T. gambieri* of Niau Island in the Tuamotu archipelago. Each of these lineages is minimally divergent in mitochondrial DNA (< 1% uncorrected P), but they are allopatric with fixed plumage differences. Sister to this clade is *T. godeffroyi* from the Marquesas Islands.

A large radiation from central Polynesia (clade D) is sister to the eastern Polynesian clade C. This radiation is geographically centered on Fiji, but extends west to Makira and Rennell Islands in the Solomon Islands and east to Tonga and American Samoa, to the exclusion of “Western” Samoa. Numerous island- or archipelago-specific lineages were monophyletic, but many basal relationships were equivocal in clade D. Geographic differentiation was evident with clades from Vanuatu, Tonga, American Samoa, the eastern Solomon Islands, and Fiji. Perhaps the most novel finding in this clade involved a biogeographic break in the eastern Solomon Islands between Guadalcanal and Makira Islands. Thus, clade D was defined as lineages occurring east of this line (e.g., *T. c. solomonis*, Makira and Ugi; *T. c. amoenus*, Rennell; and *T. c. ornatus*, Santa Cruz group). Lineages to the west (i.e., the main Solomon Islands chain plus the New Georgia group) formed a distinctly unrelated monophyletic group in clade I (Fig. 2). Thus, two clades that span thousands of kilometers of the Pacific are separated by a 60-km water gap. This biogeographic break in the eastern Solomon Islands is not novel—several other taxa exhibit breaks there including the *Monarcha castaneiventris* complex [5], *Pachycephala orioloides* [6, 7], and *Ptilinopus viridis*/*P. euganiae* [8]; however, this break generally splits taxa into sister groups. We are not aware of examples where this break is so profound such that taxa on either side are as divergent as possible in the phylogeny. Several additional taxa in clade D are sometimes incorrectly treated as members of an expanded *T. sanctus* [*T. c. vitiensis* and *T. c. eximius*; 4, 9].

(b) Species limits and species concepts

There is a long history of debate over species concepts in systematic ornithology [10-20] and the merits and utility of subspecies as a meaningful taxonomic rank [10-12]. The long-standing, but recently eroding, viewpoint of the ‘biological species’ has left the taxonomy of polytypic insular species complexes severely over-lumped, because direct observation of reproductive isolation could not be observed in allopatric insular populations [21]. Gill [22] argued cogently in favor of a new null hypothesis: that “distinct and reciprocally monophyletic sister populations of birds exhibit essential reproductive isolation and would not interbreed freely if they were to occur in sympatry.” Put succinctly, he argues in favor of splitting, not lumping, differentiated allopatric taxa. Gill effectively reverses the long-held burden of proof on the researcher to determine whether allopatric taxa would interbreed if given the chance. Instead, he argues that differentiated allopatric taxa should be assumed unlikely, if not incapable, of interbreeding; thus, allospecies should be treated as distinct species unless proven otherwise [22]. *Todiramphus* offers a unique opportunity to evaluate species limits in a polytypic insular species complex. Assumptions of interbreeding need not be made because multiple sympatric ingroup taxa make it possible to evaluate the result of divergence in spite of recent secondary sympatry.

Here, we follow a lineage-based species concept to evaluate species limits in the *Todiramphus chloris* species complex. We draw upon multiple lines of evidence including 1) our molecular phylogeny, 2) results of bGMYC species delimitation of the mtDNA data, 3) patterns of sympatry between multiple pairs of ingroup taxa (discussed above), and 4) knowledge of fixed plumage and/or ecological differences. It is worth recalling that despite our robust sampling, we still lacked six *Todiramphus* species (not traditionally placed in the *T. chloris* complex) and 28 of 50 nominal subspecies of *T. chloris*. Most species we lacked are Wallacean endemics, plus *T. albonotatus* from New Britain, and most subspecific diversity of *T. chloris* we lacked was from Indonesia, the Indian Ocean, and Vanuatu; thus, we recommend that preliminary taxonomic treatment be considered with caution.

*Syma* is the sister lineage to *Todiramphus*. *Todiramphus nigrocyaneus* is the first species to branch in the genus, and it is 11.9% diverged (ND2 uncorrected P) from *Syma*. Three phenotypically distinct populations of *T. nigrocyaneus* are distributed across New Guinea: *T. n. nigrocyaneus*, *T. n. quadricolor*, and *T. n. stictolaemus*, of which we sampled the latter from southern Papua New Guinea. This group warrants further phylogeographic study to include all three nominal subspecies of *T. nigrocyaneus*.

*Todiramphus winchelli*, *T. pyrrhopygius*, *T. macleayii*, *and T. leucopygius* are unequivocally considered valid species by taxonomists, and our study supports this treatment. All but *T. pyrrhopygius* form a group of morphologically cohesive species defined by deep blue upperparts, which is different from the blue-green typical of other *Todiramphus* species. Some authors have included *T. diops*, *T. lazuli*, *T. funebris*, *T. albonotatus*, and *T. farquhari* in this morpho-group [2], but, given limited sampling, our results suggest there is no phylogenetic basis for such a grouping. Indeed, *T. farquhari* of Vanuatu is closely allied with the *T. chloris* ingroup, whereas the other species’ affinities and genetic distinctiveness remain uncertain. Two additional species, *T. australasia* and *T. enigma* are thought to be closely allied with *T. sanctus* and *T. chloris*, respectively, based on phenotypic similarities; however, little can be said of their relationships because we lacked samples. Continued efforts to collect specimens with associated genetic material is necessary to include these six species in an expanded *Todiramphus* phylogeny. Until then, any speculation as to their placement should be treated cautiously.

(c) Proposed taxonomic revision

Species limits of ingroup clade A (Fig. 3) are complex and in need of major revision. Our phylogenetic results highlight numerous clades that warrant species status. Results of a bGMYC species delimitation analysis suggested the presence of 26 species in clade A (Fig. 3). This liberal interpretation is based on population-level sampling of mtDNA sequences only, but it provides one metric for comparison to other lineages. Below we provide an annotated list of a ‘middle ground’ approach to species delimitation (n=19 ingroup species) with comments on their relative divergence, fixed phenotypic and ecological characters, and patterns of sympatry between congeners.

- ***Todiramphus farquhari* Sharpe, 1899 (Vanuatu Kingfisher).** Unequivocally considered a valid biological species by all authors. It is as morphologically distinct as any ingroup lineage. Thought by some to be part of the dark blue-and-white morpho-group [2], but our results support a close affinity with the ingroup.

The Micronesian endemic *T. cinnamominus* has three extant nominal subspecies that are distributed on Palau, Guam, and Pohnpei. A fourth taxon, *T. cinnamominus miyakoensis*, is known from only one specimen, thought to be from the Ryukyu Islands, Japan, but its locality is uncertain [2, 23]. This taxon, if valid, is presumed extinct. All three extant *T. cinnamominus* taxa differ substantially in plumage and size, and are highly allopatric from each other. Our molecular data show that *T. cinnamominus pelewensis* of Palau is well differentiated from the other *T. cinnamominus*, but its phylogenetic placement is equivocal. The MrBayes analysis placed it inside the ingroup, whereas the BEAST analysis placed it just outside. Neither case was well supported, and both analyses recovered short internode distances, suggesting an uncertain evolutionary history of this taxon. The remaining two taxa, *T. cinnamominus cinnamominus* of Guam and *T. cinnamominus reichenbachii* of Pohnpei appear to be closely related, albeit paraphyletic with respect to *T. recurvirostris*, a Samoan endemic species. This clade represents a biogeographic enigma with three geographically disparate island distributions spanning Micronesia and Central Polynesia. We recommend species status for the three *T. cinnamominus* taxa because they are not each others’ closest relatives and there are fixed phenotypic differences, as well as vast distances of open ocean between their respective Micronesian distributions (though extinction could mask a formerly more widespread taxon). Recently, these taxa were split into three species based on phenotypic characters [24].

- ***Todiramphus pelewensis* Wiglesworth, 1891 (Rusty-capped Kingfisher).** This taxon is sympatric with *T. chloris teraokai* and differs substantially from it and other *T. cinnamominus* forms in size, plumage, and habitat preference. We follow Pratt and Etpison [25] in their use of Rusty-capped Kingfisher for an English name.
- ***Todiramphus cinnamominus* Swainson, 1821 (Guam Kingfisher).** The nominal *T. cinnamominus* from Guam is extirpated in the wild and survives only in captive breeding programs [26]. It differs morphologically from *T. pelewensis* and *T. reichenbachii* in being entirely rufous below, whereas rufous is confined to the crown of the other two species. Genetically, it is 2% diverged (ND2 uncorrected P) from *T. pelewensis*, but only 0.01% diverged from *T. reichenbachii*.
- ***Todiramphus reichenbachii* Hartlaub, 1852 (Pohnpei Kingfisher).** Endemic to Pohnpei, Caroline Islands. See *T. cinnamominus* and *T. recurvirostris* discussions for details. There is evidence for cooperative breeding in *T. reichenbachii* [27], but this behavior is also known to occur in other Pacific *Todiramphus* [e.g., *T. veneratus youngi* and *T. ruficollaris*; 28, 29, 30]. Additional comparative study of other *Todiramphus* kingfishers is needed to place cooperative breeding into phylogenetic context.
- ***Todiramphus recurvirostris* Lafresnaye, 1842 (Flat-billed Kingfisher).** This species is sister to *T. reichenbachii*. Authors have variously treated *T. recurvirostris* as its own species or as part of *T. sanctus* [2, 3]. Our results warrant species status based on its phylogenetic differentiation from *T. sanctus* and morphological differences including small size and bill morphology. It is endemic to Samoa, where it is the only *Todiramphus*; however, it is absent from American Samoa, where it is replaced by *T.* [*chloris*] *sacer* (see below).

The following three species form a clade centered on Australia and New Guinea. Within this clade, three lineages were recovered in our phylogenetic analysis, and bGMYC species delimitation supported all three as species, as well. Relationships between the lineages, however, were equivocal. Further sampling is needed, especially in the China Straight, to better understand species limits in this clade. We recommend that all three lineages be recognized as species.

- ***Todiramphus colonus* Hartert, 1896 (Colonist Kingfisher).** Breeds on islands off southeast New Guinea coast, including D’Entrecasteaux and Louisiade Archipelagos. Plumage of upperparts is brighter blue-green, which differs from the darker, more dusky upperparts of *T. sordidus*. *Todiramphus colonus* is up to 60% smaller than *T. sordidus* in body mass and differs in morphometrics, as well [31].
- ***Todiramphus sordidus* Gould, 1842 (Mangrove Collared Kingfisher).** Breeds in coastal Australia. This species likely includes nominal subspecies *sordidus*, *pilbara*, and *colcloughi.* Further sampling is recommended to better understand the phylogeographic history of these forms in Australia. Of particular interest is *pilbara* of Western Australia, which is phenotypically different from other Australian taxa [32], but was not sampled in this study.
- ***Todiramphus sanctus* Vigors & Horsfield, 1827 (Sacred Kingfisher).** Breeds throughout Australia, New Zealand, and several Melanesian islands where the full extent of its breeding range is not fully understood. Breeding is known from Kolombangara, Guadalcanal, and Three Sisters Islands in the Solomon Islands [33], and it is resident in the Santa Cruz group, Solomon Islands, parts of Vanuatu, and New Caledonia, including the Loyalty Islands. Elsewhere, it is migratory throughout Melanesia to Vanuatu and westward through New Guinea and insular southeast Asia. Throughout its breeding range, it is sympatric with multiple *T. chloris* taxa, as well as several other species including *T. pyrrhopygius*, *T. leucopygius*, *and T. farquhari* (Fig. 3). Some authors expanded the taxonomic scope of *T. sanctus* with respect to *T. chloris* and *T. recurvirostris* to include as many as nine nominal subspecies [2-4, 9]. Pratt [4] attributed the Fijian populations, *T. c. vitiensis* and *T. c. eximius*, as part of *T. sanctus* based on plumage and voice. *Todiramphus recurvirostris* from Upolu and Savai’i, Samoa is sometimes lumped as part of *T. sanctus* because differences in bill morphology are minimal [2, 3]. Our results support a more restricted circumscription of *T. sanctus*. Furthermore, we found no evidence for geographic differentiation between the three nominal subspecies sampled, suggesting ongoing gene flow—possibly aided by their migratory nature.

A large radiation across southeast Asia is represented by clade H. The basal lineage is the nominal subspecies *T. c. chloris*, which is widespread throughout Wallacea (sampled here from Sulawesi). Samples from Singapore comprise another lineage (*T. c. humii*), which is, in turn, sister to a large clade from the Philippines, Borneo, and Palau. Interestingly, despite the geographic complexity of the Philippines, no genetic structure was found across the entire archipelago. Lack of biogeographic structure in Philippines birds has been found in *Rhipidura javanica* [34] and *Copsychus saularis* [35], but the majority of terrestrial vertebrates in the Philippines show extensive genetic differentiation across and within islands [36-44]. The Palau result is completely novel in birds and further investigation should be taken to determine the origins of Palau’s avifauna. Despite the relative proximity between Palau and the Mariana Islands, this result highlights their different geologic histories in belonging to different island arc systems.

- ***Todiramphus chloris* Boddaert, 1783 (Collared Kingfisher).** We support a conservative approach by treating the large Asian clade H as one species. We recognize that there is genetic structure in this clade worthy of further species delimitation (i.e., Sulawesi; mainland southeast Asia; and Borneo, Philippines, and Palau), but there are too many gaps in our sampling to say definitively.

In clade I, we recommend recognizing three species. Further sampling is necessary in the Bismarck Archipelago (*T. albonotatus* of New Britain, and several nominal subspecies of the *T. chloris* complex from Musau (*matthiae*) to Nissan (*bennetti*).

- ***Todiramphus saurophagus* Gould, 1843 (Beach Kingfisher).** The largest *Todiramphus* species. This coastal specialist is distinctive morphologically with a massive bill and white head.
- ***Todiramphus albicilla* Dumont, 1823 (Mariana Kingfisher).** The sister species to *T. saurophagus*. Our sampling is incomplete, so we treat this recommendation with caution. We sampled birds from Saipan (*albicilla*) and Rota (*orii*), but lacked samples from Asuncion, Agrihan, Pagan, and Almagan in the northern part of the archipelago (*owstoni*). All forms are large, like *T. saurophagus*, but only *albicilla* from Saipan is white-headed. The other forms are variably white or blue-crowned suggesting this trait is phenotypically plastic in this clade. Interestingly, birds from Mussau Island in the St. Matthias Islands, the northernmost island in the greater Bismarck Archipelago is similarly plumaged to *orii* and *owstoni*. Furthermore, about 40% of individuals of *T. saurophagus admiralitatis* from the Admiralty Islands show blue-green crowns [33]. This pattern is suggestive of ancestral polymorphisms of crown plumage within the broader clade of *T. saurophagus* + *T. albicilla*.
- ***Todiramphus tristrami* E. L. Layard, 1880 (Melanesian Kingfisher).** This species corresponds to a geographically cohesive clade from the Bismarck Archipelago and the main Solomon Islands chain (Bougainville to Guadalcanal). We sampled only two nominal subspecies (*nusae* and *alberti*), but *tristrami* (New Britain) has priority. Much denser sampling is needed including the following taxa: *mathiae*, *stresemanni*, *novaehiberniae*, *bennetti*, *tristrami*, and *pavuvu*.

Clade D contains numerous lineages endemic to relatively small geographic areas in central Polynesia. The bGMYC species delimitation results supports seven species (Fig. 3). We caution against this interpretation given the unresolved topology and large number of sampling gaps from this region. For example, Mayr examined the kingfishers of central Polynesia, which resulted in his naming 15 nominal subspecies of *T. chloris* [45-48], of which we sampled 10. Thus, we treat clade D as one polytypic species, and recommend this clade for further study with improved geographic sampling, especially from the clade’s geographic center: Vanuatu.

- ***Todiramphus sacer* J. F. Gmelin, 1788 (Pacific Kingfisher).** A widespread species endemic to central Polynesia from the eastern Solomon Islands (Makira, Rennell, Santa Cruz group, and possibly Malaita [unsampled]), Vanuatu, Fiji, Tonga, and American Samoa (absent from Samoa where it is replaced by *T. recurvirostris*).

Clade C comprises a radiation of eastern Polynesian kingfishers that have long-been treated as 5–6 species [2, 3, 23]. Our phylogenetic and species delimitation results support this at most nodes. We recommend maintaining current taxonomy of the following five phenotypically and genetically differentiated species [49].

- ***Todiramphus godeffroyi* Finsch, 1877 (Marquesas Kingfisher).** Endemic to the Marquesas Islands.
- ***Todiramphus ruficollaris* Holyoak, 1974 (Mewing Kingfisher).** Endemic to the Cook Islands.
- ***Todiramphus veneratus* J. F. Gmelin, 1788 (Society Kingfisher).** Endemic to the Windward Society Islands. Two nominal subspecies are described (*T. v. veneratus* on Tahiti and *T. v. youngi* on Moorea). We found no support for reciprocal monophyly of these forms, and *T. v. veneratus* was only 0.19% diverged from *T. v. youngi* (ND2 uncorrected P). This genetic difference is higher than that between *T. cinnamominus* and *T. reichenbachii* (0.01%), which we argue for species status not based on absolute genetic difference, but rather on the monophyly of the clades. Furthermore, the plumage differences between *T. v. veneratus* and *T. v. youngi* are stark: The blue dorsal color of *T. v. veneratus* is replaced with brown in *T. v. youngi*, a rare color in *Todiramphus* kingfishers [2]. We recommend further study of these kingfishers to examine in detail their evolutionary history, biogeography, and species limits.
- ***Todiramphus gertrudae* Murphy, 1924 (Niau Kingfisher).** Endemic to Niau, Tuamotu Archipelago. Sometimes treated as conspecific with *T. gambieri*, but see below for further discussion.
- ***Todiramphus gambieri* Oustalet, 1895 (Tuamotu Kingfisher).** Known only by the type specimen collected on Mangareva, Gambier Is. in 1838. It differs from *T. gertrudae* by several distinctive plumage traits [50]. Taking into account both distance separating Niau and Gambier Islands (>1,400 km) and their respective geological histories, we recommend treating *T. gertrudae* and *T. gambieri* as separate species. Our attempt to obtain DNA from the specimen failed.
- ***Todiramphus tutus* J. F. Gmelin, 1788 (Chattering Kingfisher).** Widespread in the Cook and Leeward Society Islands. We sampled all three nominal subspecies (*T. t. tutus*, *T. t. atiu*, and *T. t. mauke*), but there was no support for reciprocal monophyly of the three.

References

[1] Steadman, D.W. 2006 *Exctinction and Biogeography of Tropical Pacific Birds*. Chicago, IL, University of Chicago Press; 594 p.

[2] Woodall, P.F. 2001 Family Alcedinidae (kingfishers). In *Handbook of the Birds of the World. Vol. 6. Mousebirds to hornbills* (eds. J. del Hoyo, A. Elliott & J. Sargatal), pp. 130–249. Barcelona, Spain, Lynx Edicions.

[3] Fry, C.H. 1980 The evolutionary biology of kingfishers (Alcedinidae). *Living Bird.* **18**, 113–160.

[4] Pratt, H.D. 1987 *A Field Guide to the Birds of Hawaii and the Tropical Pacific*. Princeton, New Jersey, Princeton University Press.

[5] Uy, J.A.C., Moyle, R.G. & Filardi, C.E. 2009 Plumage and song differences mediate species recognition between incipient flycatcher species of the Solomon Islands. *Evolution.* **63**, 153–164. (doi:10.1111/j.1558-5646.2008.00530.x).

[6] Andersen, M.J., Nyári, Á.S., Mason, I., Joseph, L., Dumbacher, J.P., Filardi, C.E. & Moyle, R.G. 2014 Molecular systematics of the world's most polytypic bird: the *Pachycephala pectoralis*/*melanura* (Aves: Pachycephalidae) species complex. *Zool. J. Linn. Soc.* **170**, 566–588. (doi:10.1111/zoj.12088).

[7] Jønsson, K.A., Irestedt, M., Christidis, L., Clegg, S.M., Holt, B.G. & Fjeldsa, J. 2014 Evidence of taxon cycles in an Indo-Pacific passerine bird radiation (Aves: *Pachycephala*). *Proceeding of the Royal Society of London Series B* **281**, 20131727. (doi:10.1098/rspb.2013.1727).

[8] Cibois, A., Thibault, J.-C., Bonillo, C., Filardi, C.E., Watling, D. & Pasquet, E. 2014 Phylogeny and biogeography of the fruit doves (Aves: Columbidae). *Mol. Phylogenet. Evol.* **70**, 442–453. (doi:http://dx.doi.org/10.1016/j.ympev.2013.08.019).

[9] Clements, J.F., Schulenberg, T.S., Iliff, M.J., Sullivan, B.L., Wood, C.L. & Roberson, D. 2013 The eBird/Clements Checklist of Birds of the World: Version 6.8. (

[10] Remsen, J.J.V. 2010 Subspecies as a meaningful taxonomic rank in avian classification. *Ornithol. Monogr.* **67**, 62–78. (doi:doi/10.1525/om.2010.67.1.62).

[11] Fitzpatrick, J.W. 2010 Subspecies are for convenience. *Ornithol. Monogr.* **67**, 54–61. (doi:doi/10.1525/om.2010.67.1.54).

[12] Pratt, H.D. 2010 Revisiting species and subspecies of island birds for a better assessment of biodiversity. *Ornithol. Monogr.* **67**, 78–89. (doi:doi/10.1525/om.2010.67.1.79).

[13] Cracraft, J. 1989 Speciation and its ontology: the empirical consequences of alternative species concepts for understanding patterns and processes of differentiation. In *Speciation and its Consequences* (eds. D. Otte & J. Endler), pp. 28–59. Sunderland, MA, Sinauer Associates.

[14] Cracraft, J. 1983 Species concepts and speciation analysis. *Curr. Ornithol.* **1**, 159–187.

[15] de Queiroz, K. 1999 The general lineage concept of species and the defining properties of the species category. In *Species: New Interdisciplinary Essays* (ed. R.A. Wilson), pp. 46–89. Cambridge, Massachusetts, A Bradford Book.

[16] de Queiroz, K. 1998 The general lineage concept of species, species criteria, and the process of speciation: a conceptural unification and terminological recommendations. In *Endless Forms: Species and Speciation* (eds. D.J. Howard & S.H. Berlocher), pp. 57–75. Oxford, U.K., Oxford University Press.

[17] Remsen, J.V. 2005 Pattern, process, and rigor meet classification. *Auk.* **122**, 403–413.

[18] Zink, R.M. 2006 Rigor and species concepts. *Auk.* **123**, 887–891. (doi:doi/10.2307/4090565?ref=search-gateway:568f24d96d166e37613e107073653af3).

[19] Zink, R.M. & McKitrick, M.C. 1995 The debate over species concepts and its implications for ornithology. *Auk.* **112**, 701–719. (doi:doi/10.2307/4088685?ref=search-gateway:568f24d96d166e37613e107073653af3).

[20] McKitrick, M.C. & Zink, R.M. 1988 Species concepts in ornithology. *Condor.* **90**, 1–14.

[21] Mayr, E. & Diamond, J. 2001 *The Birds of Northern Melanesia: Speciation, Ecology, and Biogeography*. New York, Oxford University Press.

[22] Gill, F.B. 2014 Species taxonomy of birds: which null hypothesis? *Auk.* **131**, 150–161. (doi:doi/10.1642/AUK-13-206.1).

[23] Fry, C.H., Fry, K. & Harris, A. 1992 *Kingfishers, Bee-eaters & Rollers: A handbook*. Princeton, New Jersey, Princeton University Press; 324 p.

[24] del Hoyo, J., Collar, N.J., Christie, D.A., Elliot, A. & Fishpool, L.D.C. 2014 *HBW and BirdLife International Illustrated Checklist of the Birds of the World. Volume 1: Non-passerines.* Barcelona, Spain and Cambridge, UK, Lynx Edicions and BirdLife International.

[25] Pratt, H.D. & Etpison, M.T. 2008 *Birds and Bats of Palau*. Honolulu, HI, Mutual Publishing, L.L.C.

[26] Haig, S.M. & Ballou, J.D. 1995 Genetic diversity in two avian species formerly endemic to Guam. *Auk.* **112**, 445–455. (doi:doi/10.2307/4088732?ref=search-gateway:485e7b60fa748053e6e467078b12847a).

[27] Kesler, D.C. & Haig, S.M. 2007 Territoriality, prospecting, and dispersal in cooperatively breeding Micronesian kingfishers (*Todiramphus cinnamominus reichenbachii*). *Auk.* **124**, 381–395. (doi:10.1642/0004-8038(2007)124[381:tpadic]2.0.co;2).

[28] Kesler, D.C., Ghestemme, T., Portier, E. & Gouni, A. 2010 Cooperative breeding of the Society kingfisher (*Todiramphus veneratus*). *Wilson J. Ornithol.* **122**, 46–50.

[29] Holyoak, D.T. & Thibault, J.C. 1984 Contribution à l'étude des oiseaux de Polynésie orientale. *Memoires du Museum National d'Histoire Naturelle - Serie A: Zoologie* **127**, 1–209.

[30] Rowe, S. & Empson, R. 1996 Distribution and abundance of the Tanga'eo or Mangaia Kingfisher (Halcyon tuta ruficollaris). *Notornis* **43**, 35–42.

[31] Forshaw, J.M. & Cooper, W.T. 1985 *Kingfishers and Related Birds Vol. 2: Alcedinidae Halcyon to Tanysiptera*. Sydney, Lansdowne Editions.

[32] Johnstone, R.E. & Storr, G.M. 1998 *Handbook of Western Australian Birds, Volume 1: Non-passerines (Emu to Dollarbird)*. Perth, Australia, Western Australian Museum; 436 p.

[33] Dutson, G., Allen, R., Bowley, A., Cox, J. & Disley, T. 2011 *Birds of Melanesia, Bismarcks, Solomons, Vanuatu, and New Caledonia*. Princeton, New Jersey, Princeton University Press; 447 p.

[34] Sánchez-González, L.A. & Moyle, R.G. 2011 Molecular systematics and species limits in the Philippine fantails (Ayes: *Rhipidura*). *Mol. Phylogenet. Evol.* **61**, 290–299. (doi:10.1016/j.ympev.2011.06.013).

[35] Sheldon, F.H., Lohman, D.J., Lim, H.C., Zou, F., Goodman, S.M., Prawiradilaga, D.M., Winker, K., Braile, T.M. & Moyle, R.G. 2009 Phylogeography of the magpie-robin species complex (Aves: Turdidae: *Copsychus*) reveals a Philippine species, an interesting isolating barrier, and unusual dispersal patterns in the Indian Ocean and Southeast Asia. *J. Biogeogr.* **36**, 1070–1083.

[36] Brown, R.M., Siler, C.D., Oliveros, C.H., Esselstyn, J.A., Diesmos, A.C., Hosner, P.A., Linkem, C.W., Barley, A.J., Oaks, J.R., Sanguila, M.B., et al. 2013 Evolutionary processes of diversification in a model island archipelago. *Annu. Rev. Ecol. Evol. Syst.* **44**, 411–435. (doi:doi/10.1146/annurev-ecolsys-110411-160323).

[37] Barley, A.J., White, J., Diesmos, A.C. & Brown, R.M. 2013 The challenge of species delimitation at the extremes: diversification without morphological change in Philippine sun skinks. *Evolution.* **67**, 3556–3572. (doi:doi/10.1111/evo.12219).

[38] Linkem, C.W., Siler, C.D., Diesmos, A.C., Sy, E. & Brown, R.M. 2010 A new species of Gekko (Squamata: Gekkonidae) from central Luzon Island, Philippines. *Zootaxa* **2396**, 37–49.

[39] Hosner, P.A., Nyári, Á.S. & Moyle, R.G. 2013 Water barriers and intra-island isolation contribute to diversification in the insular *Aethopyga* sunbirds (Aves: Nectariniidae). *J. Biogeogr.* **40**, 1094–1106. (doi:doi:10.1111/jbi.12074).

[40] Linkem, C.W., Hesed, K.M., Diesmos, A.C. & Brown, R.M. 2010 Species boundaries and cryptic lineage diversity in a Philippine forest skink complex (Reptilia; Squamata; Scincidae: Lygosominae). *Mol. Phylogenet. Evol.* **56**, 572–585. (doi:10.1016/j.ympev.2010.03.043).

[41] Oliveros, C.H. & Moyle, R.G. 2010 Origin and diversification of Philippine bulbuls. *Mol. Phylogenet. Evol.* **54**, 822–832. (doi:10.1016/j.ympev.2009.12.001).

[42] Hosner, P.A., Boggess, N.C., Alviola, P., Sánchez-González, L.A., Oliveros, C.H., Urriza, R. & Moyle, R.G. 2013 Phylogeography of the *Robsonius* ground-warblers (Passeriformes: Locustellidae) reveals an undescribed species from northeastern Luzon, Philippines. *Condor.* **115**, 630–639. (doi:doi/10.1525/cond.2013.120124).

[43] Sheldon, F.H., Oliveros, C.H., Taylor, S.S., McKay, B., Lim, H.C., Rahman, M.A., Mays, H. & Moyle, R.G. 2012 Molecular phylogeny and insular biogeography of the lowland tailorbirds of Southeast Asia (Cisticolidae: *Orthotomus*). *Mol. Phylogenet. Evol.* **65**, 54–63. (doi:10.1016/j.ympev.2012.05.023).

[44] Esselstyn, J.A. & Brown, R.M. 2009 The role of repeated sea-level fluctuations in the generation of shrew (Soricidae: *Crocidura*) diversity in the Philippine Archipelago. *Mol. Phylogenet. Evol.* **53**, 171–181. (doi:Doi 10.1016/J.Ympev.2009.05.034).

[45] Mayr, E. 1931 Birds collected during the Whitney South Sea Expedition, 12. Notes on *Halcyon chloris* and some of its subspecies. *Am. Mus. Novit.* **469**, 1–10.

[46] Mayr, E. 1935 Birds collected during the Whitney South Sea Expedition, 30. Twenty-four apparently undescribed birds from New Guinea and the D'Entrecasteaux Archipelago. *Am. Mus. Novit.* **814**, 1–6. (doi:uuid/9C6A8FCD-E726-4FC7-8ACD-336744381DDD).

[47] Mayr, E. 1938 Birds collected during the Whitney South Sea Expedition, 38. On a collection from Erromanga, New Hebrides. *Am. Mus. Novit.* **986**, 1–3. (doi:uuid/936EB90C-1E0B-42BF-A199-417CC493AF13).

[48] Mayr, E. 1941 Birds collected during the Whitney South Sea Expedition, 47. Notes on the genera *Halcyon*, *Turdus*, and *Eurostopodus*. *Am. Mus. Novit.* **1152**, 1–7. (doi:uuid/AFABB997-EE07-4B70-89F5-C3271A16CDF5).

[49] Gill, F.B. & Donsker, D. 2014 IOC World Bird List (v 4.1). Available at http://www.worldbirdnames.org. (

[50] Holyoak, D.T. & Thibault, J.-C. 1977 *Halcyon gambieri gambieri* Oustalet, an extinct kingfisher from Mangareva, South Pacific Ocean. *Bull. Brit. Ornithol. Club* **97**, 21–23.

[51] Backström, N., Fagerberg, S. & Ellegren, H. 2008 Genomics of natural bird populations: a gene-based set of reference markers evenly spread across the avian genome. *Molecular Ecology* **17**, 964–980. (doi:10.1111/j.1365-294X.2007.03551.x).

[52] Kimball, R.T., Braun, E.L., Barker, F.K., Bowie, R.C.K., Braun, M.J., Chojnowski, J.L., Hackett, S., Han, K.-L., Harshman, J., Heimer-Torres, V., et al. 2009 A well-tested set of primers to amplify regions spread across the avian genome. *Mol. Phylogenet. Evol.* **50**, 654–660.

[53] Primmer, C.R., Borge, T., Lindell, J. & Sætre, G.P. 2002 Single-nucleotide polymorphism characterization in species with limited available sequence information: high nucleotide diversity revealed in the avian genome. *Molecular Ecology* **11**, 603–612. (doi:Doi 10.1046/J.0962-1083.2001.01452.X).

[54] Johnson, K.P., McKinney, F. & Sorenson, M.D. 1999 Phylogenetic constraint on male parental care in the dabbling ducks. *Proceeding of the Royal Society of London Series B* **266**, 759–763.

Table captions

Table ESM1 (Electronic Supplementary Material). List of samples used in the study following the taxonomy of [49]. Ancient DNA samples derived from museum specimens (i.e., toepads), unvouchered blood samples, and samples used in BEAST analyses are noted with superscripts. Institutional abbreviations: AMNH, American Museum of Natural History; ANWC, Australian National Wildlife Collection; FMNH, Field Museum of Natural History; KUNHM, University of Kansas Natural History Museum; LSUMNS, Louisiana State University Museum of Natural Science; MHNG, Muséum d'histoire naturelle de la Ville de Genève; MNHN, Le Muséum National d'Histoire Naturelle; SNZP, Smithsonian National Zoological Park; UWBM, University of Washington Burke Museum.

Table ESM2 (Electronic Supplementary Material). Newly-designed primers to sequence samples derived from museum specimen toe pads.

Table ESM3 (Electronic Supplementary Material). Summary statistics of the six gene regions sequenced in this study.

Table ESM1 (Electronic Supplementary Material).

| genus | species | subspecies | institution | sample | locality |
| --- | --- | --- | --- | --- | --- |
| Ingroup |  |  |  |  |  |
| *Todiramphus* | *chloris* | *alberti* | UWBM | Bu60188 | SOLOMON ISLANDS: Isabel Is. |
| *Todiramphus* | *chloris* | *alberti* | UWBM | Bu60266 | SOLOMON ISLANDS: Guadalcanal Is. |
| *Todiramphus* | *chloris* | *alberti* | UWBM | Bu60296 | SOLOMON ISLANDS: Kiaba Is. (north coast Isabel Is.) |
| *Todiramphus* | *chloris* | *alberti* | UWBM | Bu60320 | SOLOMON ISLANDS: Fera Is. (north coast Isabel Is.) |
| *Todiramphus* | *chloris* | *alberti*^B^ | UWBM | Bu60362 | SOLOMON ISLANDS: Guadalcanal Is. |
| *Todiramphus* | *chloris* | *alberti* | UWBM | Bu63065 | SOLOMON ISLANDS: Choiseul Is. |
| *Todiramphus* | *chloris* | *alberti* | UWBM | Bu63233 | SOLOMON ISLANDS: Choiseul Is. |
| *Todiramphus* | *chloris* | *alberti*^B^ | UWBM | Bu66007 | SOLOMON ISLANDS: New Georgia Is. |
| *Todiramphus* | *chloris* | *alberti* | UWBM | Bu66038 | SOLOMON ISLANDS: New Georgia Is. |
| *Todiramphus* | *chloris* | *alberti* | AMNH | DOT6704 | SOLOMON ISLANDS: Guadalcanal Is. |
| *Todiramphus* | *chloris* | *albicilla*^B^ | KUNHM | 22581 | NORTHERN MARIANA ISLANDS: Saipan Is. |
| *Todiramphus* | *chloris* | *albicilla* | KUNHM | 22591 | NORTHERN MARIANA ISLANDS: Saipan Is. |
| *Todiramphus* | *chloris* | *albicilla*^B^ | KUNHM | 22592 | NORHTERN MARIANA ISLANDS: Saipan Is. |
| *Todiramphus* | *chloris* | *albicilla* | KUNHM | 22603 | NORTHERN MARIANA ISLANDS: Saipan Is. |
| *Todiramphus* | *chloris* | *albicilla* | KUNHM | 22611 | NORTHERN MARIANA ISLANDS: Saipan Is. |
| *Todiramphus* | *chloris* | *amoenus*^B^ | UWBM | Bu58741 | SOLOMON ISLANDS: Rennell Is. |
| *Todiramphus* | *chloris* | *amoenus* | UWBM | Bu58743 | SOLOMON ISLANDS: Rennell Is. |
| *Todiramphus* | *chloris* | *amoenus*^B^ | AMNH | DOT6588 | SOLOMON ISLANDS: Rennell Is. |
| *Todiramphus* | *chloris* | *chloris*^B^ | AMNH | DOT12606 | INDONESIA: Sulawesi Is. |
| *Todiramphus* | *chloris* | *collaris* | KUNHM | 13960 | PHILIPPINES: Camiguin Sur Is. |
| *Todiramphus* | *chloris* | *collaris* | KUNHM | 13971 | PHILIPPINES: Camiguin Sur Is. |
| *Todiramphus* | *chloris* | *collaris* | KUNHM | 14010 | PHILIPPINES: Camiguin Sur Is. |
| *Todiramphus* | *chloris* | *collaris*^B^ | KUNHM | 14446 | PHILIPPINES: Tablas Is. |
| *Todiramphus* | *chloris* | *collaris* | KUNHM | 14447 | PHILIPPINES: Tablas Is. |
| *Todiramphus* | *chloris* | *collaris* | KUNHM | 17938 | PHILIPPINES: Batan Is. |
| *Todiramphus* | *chloris* | *collaris* | KUNHM | 18130 | PHILIPPINES: Mindanao Is. |
| *Todiramphus* | *chloris* | *collaris*^B^ | KUNHM | 18134 | PHILIPPINES: Mindanao Is. |
| *Todiramphus* | *chloris* | *collaris* | KUNHM | 28455 | PHILIPPINES: Mindanao Is. |
| *Todiramphus* | *chloris* | *collaris* | KUNHM | 28674 | PHILIPPINES: Mindanao Is. |
| *Todiramphus* | *chloris* | *collaris* | KUNHM | 20983 | PHILIPPINES: Bohol Is. |
| *Todiramphus* | *chloris* | *collaris* | UWBM | F358326 | PHILIPPINES: Sibuyan Is. |
| *Todiramphus* | *chloris* | *colonus*^B^ | SNZP | TKP2003070 | PNG: Louisiade Archipelago; Rossel Is. |
| *Todiramphus* | *chloris* | *colonus* | SNZP | TKP2003071 | PNG: D’Entrecasteaux Archipelago; Duchess Is. |
| *Todiramphus* | *chloris* | *colonus*^B^ | SNZP | TKP2003089 | PNG: D’Entrecasteaux Archipelago; Tobwoiama Is. |
| *Todiramphus* | *chloris* | *colonus* | SNZP | TKP2003092 | PNG: D’Entrecasteaux Archipelago; Tobwoiama Is. |
| *Todiramphus* | *chloris* | *colonus* | SNZP | TKP2003097 | PNG: D’Entrecasteaux Archipelago; Tobwoiama Is. |
| *Todiramphus* | *chloris* | *eximius*^B^ | KUNHM | 25219 | FIJI: Kadavu Is. |
| *Todiramphus* | *chloris* | *eximius*^B^ | KUNHM | 25227 | FIJI: Kadavu Is. |
| *Todiramphus* | *chloris* | *humii*^B^ | UWBM | Bu67535 | SINGAPORE |
| *Todiramphus* | *chloris* | *humii*^B^ | UWBM | Bu76183 | SINGAPORE |
| *Todiramphus* | *chloris* | *humii* | UWBM | Bu76211 | SINGAPORE |
| *Todiramphus* | *chloris* | *laubmannianus*^B^ | UWBM | Bu81948 | MALAYSIA: Borneo; Sarawak |
| *Todiramphus* | *chloris* | *manuae* ^†^ | KUNHM | 104154 | AMERICAN SAMOA: Ta‘ū Is. |
| *Todiramphus* | *chloris* | *manuae* ^†^ | KUNHM | 104156 | AMERICAN SAMOA: Ofu Is. |
| *Todiramphus* | *chloris* | *manuae* ^†B^ | KUNHM | 104157 | AMERICAN SAMOA: Ta‘ū Is. |
| *Todiramphus* | *chloris* | *manuae* ^†B^ | KUNHM | 107630 | AMERICAN SAMOA: Ofu Is. |
| *Todirampus* | *chloris* | *marinus*^B^ | KUNHM | 26338 | FIJI: Lau Archipelago; Ogea Levu Is. |
| *Todiramphus* | *chloris* | *marinus* | KUNHM | 26342 | FIJI: Lau Archipelago; Ogea Driki Is. |
| *Todiramphus* | *chloris* | *marinus* | KUNHM | 26348 | FIJI: Lau Archipelago; Ogea Levu Is. |
| *Todiramphus* | *chloris* | *marinus* | KUNHM | 26369 | FIJI: Lau Archipelago; Namuka-i-Lau Is. |
| *Todiraphus* | *chloris* | *marinus* | KUNHM | 26383 | FIJI: Lau Archipelago; Fulaga Is. |
| *Todirampus* | *chloris* | *marinus* | KUNHM | 26393 | FIJI: Lau Archipelago; Fulaga Is. |
| *Todiramphu* | *chloris* | *marinus* | KUNHM | 26408 | FIJI: Lau Archipelago; Kabara Is. |
| *Todiramphus* | *chloris* | *marinus*^B^ | KUNHM | 26410 | FIJI: Lau Archipelago; Kabara Is. |
| *Todiramphus* | *chloris* | *marinus* | KUNHM | 26411 | FIJI: Lau Archipelago; Vuagava Is. |
| *Todiramphus* | *chloris* | *marinus* | KUNHM | 26439 | FIJI: Lau Archipelago; Vanua Vatu Is. |
| *Todiramphus* | *chloris* | *nusae*^B^ | KUNHM | 27723 | PNG: Bismarck Archipelago; New Ireland Is. |
| *Todiramphus* | *chloris* | *nusae* | KUNHM | 27753 | PNG: Bismarck Archipelago; New Ireland Is. |
| *Todirapmhus* | *chloris* | *nusae* | KUNHM | 27792 | PNG: Bismarck Archipelago; Nusalaman Is. |
| *Todiramphus* | *chloris* | *nusae* | KUNHM | 27793 | PNG: Bismarck Archipelago; Nusalaman Is. |
| *Todiramphus* | *chloris* | *nusae* | KUNHM | 27812 | PNG: Bismarck Archipelago; Nusalaman Is. |
| *Todirapmhus* | *chloris* | *nusae*^B^ | KUNHM | 27857 | PNG: Bismarck Archipelago; Dyaul Is. |
| *Todiramphus* | *chloris* | *orii*^B^ | UWBM | Bu85102 | NORTHERN MARIANA ISLANDS: Rota Is. |
| *Todiramphus* | *chloris* | *orii* | UWBM | Bu85104 | NORTHERN MARIANA ISLANDS: Rota Is. |
| *Todirampus* | *chloris* | *orii*^B^ | UWBM | Bu85105 | NORTHERN MARIANA ISLANDS: Rota Is. |
| *Todiramphus* | *chloris* | *ornatus*^B^ | KUNHM | 19404 | SOLOMON ISLANDS: Santa Cruz Group; Nendo Is. |
| *Todiramphus* | *chloris* | *pealei* ^†B^ | KUNHM | 104160 | AMERICAN SAMOA: Tutuila Is. |
| *Todiramphus* | *chloris* | *pealei* ^†^ | KUNHM | 104164 | AMERICAN SAMOA: Tutuila Is. |
| *Todiramphus* | *chloris* | *pealei*^B^ | UWBM | Bu89771 | AMERICAN SAMOA: Tutuila Is. |
| *Todiramphus* | *chloris* | *sacer*^B^ | UWBM | Bu42835 | TONGA: ‘Eua Is. |
| *Todiramphus* | *chloris* | *sacer* | UWBM | Bu42841 | TONGA: ‘Eua Is. |
| *Todiramphus* | *chloris* | *sacer*^B^ | UWBM | Bu42904 | TONGA: ‘Eua Is. |
| *Todiramphus* | *chloris* | *santoensis*^B^ | LSUMNS | B45831 | VANUATU: Santo Is. |
| *Todiramphus* | *chloris* | *solomonis*^B^ | KUNHM | 12834 | SOLOMON ISLANDS: Makira Is. |
| *Todiramphus* | *chloris* | *solomonis*^B^ | KUNHM | 15921 | SOLOMON ISLANDS: Ugi Is. (north coast Makira Is.) |
| *Todiramphus* | *chloris* | *solomonis* | KUNHM | 15922 | SOLOMON ISLANDS: Ugi Is. (north coast Makira Is.) |
| *Todiramphus* | *chloris* | *solomonis* | KUNHM | 15926 | SOLOMON ISLANDS: Ugi Is. (north coast Makira Is.) |
| *Todiramphus* | *chloris* | *sordidus*^B^ | ANWC | 33719 | AUSTRALIA: Northern Territory, NE Darwin |
| *Todiramphus* | *chloris* | *sordidus*^B^ | ANWC | 33720 | AUSTRALIA: Northern Territory, NE Darwin |
| *Todiramphus* | *chloris* | *colcloughi*^B^ | ANWC | 44296 | AUSTRALIA: Queensland; N Rockhampton |
| *Todiramphus* | *chloris* | *sordidus*^B^ | ANWC | 51462 | AUSTRALIA: Queensland; Cape York Peninsula |
| *Todiramphus* | *chloris* | *sordidus* ^†^ | KUNHM | 8589 | AUSTRALIA: Northern Territory, NE Darwin |
| *Todiramphus* | *chloris* | *teraokai*^B^ | KUNHM | 23630 | PALAU: Babeldaob Is. |
| *Todiramphus* | *chloris* | *teraokai* | KUNHM | 23631 | PALAU: Babeldaob Is. |
| *Todiramphus* | *chloris* | *teraokai*^B^ | KUNHM | 23690 | PALAU: Peleliu Is. |
| *Todiramphus* | *chloris* | *vitiensis*^B^ | KUNHM | 24247 | FIJI: Vanua Levu Is. |
| *Todiramphus* | *chloris* | *vitiensis* | KUNHM | 24248 | FIJI: Vanua Levu Is. |
| *Todiramphus* | *chloris* | *vitiensis* | KUNHM | 26496 | FIJI: Kioa Is. |
| *Todiramphus* | *chloris* | *vitiensis* | KUNHM | 26529 | FIJI: Vanua Levu Is. |
| *Todiramphus* | *chloris* | *vitiensis*^B^ | KUNHM | 30462 | FIJI: Viti Levu Is. |
| *Todiramphus* | *chloris* | *vitiensis* | KUNHM | 30469 | FIJI: Lomaiviti Group; Koro Is. |
| *Todiramphus* | *chloris* | *vitiensis* | KUNHM | 30489 | FIJI: Lomaiviti Group; Ovalau Is. |
| *Todiramphus* | *chloris* | *vitiensis* | KUNHM | 30504 | FIJI: Lomaiviti Group; Ovalau Is. |
| *Todiramphus* | *cinnamominus* | *cinnamominus*^†B^ | KUNHM | 47548 | MARIANA ISLANDS: Guam Is. |
| *Todiramphus* | *cinnamominus* | *pelewensis*^B^ | KUNHM | 23651 | PALAU: Babeldaob Is. |
| *Todiramphus* | *cinnamominus* | *pelewensis* | KUNHM | 23662 | PALAU: Babeldaob Is. |
| *Todiramphus* | *cinnamominus* | *pelewensis*^B^ | KUNHM | 23674 | PALAU: Peleliu Is. |
| *Todiramphus* | *cinnamominus* | *reichenbachii* ^†B^ | KUNHM | 40147 | MICRONESIA: Pohnpei Is. |
| *Todiramphus* | *farquhari*^B^ |  | LSUMNS | B45388 | VANUATU: Santo Is. |
| *Todiramphus* | *farquhari*^B^ |  | LSUMNS | B45401 | VANUATU: Santo Is. |
| *Todiramphus* | *gambieri* | *gertrudae*^*B^ | MHNG | PO3-43 | FRENCH POLYNESIA: Tuamotu Archipelago; Niau Is. |
| *Todiramphus* | *godeffroyi* ^†B^ |  | MNHN | 1822 | FRENCH POLYNESIA: Marquesas Archipelago; Tahuata Is. |
| *Todiramphus* | *godeffroyi* ^†B^ |  | MNHN | 1823 | FRENCH POLYNESIA: Marquesas Archipelago; Tahuata Is. |
| *Todiramphus* | *recurvirostris* ^†^ |  | KUNHM | 104171 | SAMOA: Upolu Is. |
| *Todiramphus* | *recurvirostris*^†B^ |  | KUNHM | 104172 | SAMOA: Upolu Is. |
| *Todiramphus* | *recurvirostris*^†B^ |  | KUNHM | 104178 | SAMOA: Savai‘i Is. |
| *Todiramphus* | *recurvirostris*^†^ |  | KUNHM | 104181 | SAMOA: Savai‘i Is. |
| *Todiramphus* | *ruficollaris*^B^ |  | UWBM | Bu42791 | COOK ISLANDS: Mangaia Is. |
| *Todiramphus* | *ruficollaris*^B^ |  | UWBM | Bu42806 | COOK ISLANDS: Mangaia Is. |
| *Todiramphus* | *sanctus* | *canacorum* ^*B^ | MNHN | NC10 | NEW CALEDONIA: xxxxx |
| *Todiramphus* | *sanctus* | *canacorum* ^*B^ | MNHN | NC83 | NEW CALEDONIA: xxxxx |
| *Todiramphus* | *sanctus* | *sanctus* | ANWC | 34636 | AUSTRALIA: Northern Territory; SE Darwin |
| *Todiramphus* | *sanctus* | *sanctus* | ANWC | 34659 | AUSTRALIA: Western Australia; N Albany |
| *Todiramphus* | *sanctus* | *sanctus* | ANWC | 50292 | AUSTRALIA: Western Australia; NW Mt. Barker |
| *Todiramphus* | *sanctus* | *sanctus* | ANWC | 54622 | AUSTRALIA: Northern Territory; Roper River |
| *Todiramphus* | *sanctus* | *sanctus* | KUNHM | 7557 | PNG: Western Province |
| *Todiramphus* | *sanctus* | *sanctus* | KUNHM | 7567 | PNG |
| *Todiramphus* | *sanctus* | *sanctus*^B^ | KUNHM | 19403 | SOLOMON ISLANDS: Santa Cruz Group; Nendo Is. |
| *Todiramphus* | *sanctus* | *sanctus*^B^ | LSUMNS | B45812 | VANUATU: Santo Is. |
| *Todiramphus* | *sanctus* | *sanctus*^B^ | UWBM | Bu57468 | AUSTRALIA: New South Wales |
| *Todiramphus* | *sanctus* | *sanctus* | UWBM | Bu58750 | SOLOMON ISLANDS: Santa Isabel Is. |
| *Todiramphus* | *sanctus* | *sanctus* | UWBM | Bu62818 | AUSTRALIA: New South Wales |
| *Todiramphus* | *sanctus* | *sanctus*^B^ | UWBM | Bu63200 | SOLOMON ISLANDS: Choiseul Is. |
| *Todiramphus* | *sanctus* | *sanctus* | UWBM | Bu68059 | PNG: Bismarck Archipelago; Schumann Is. (north coast New Britain Is.) |
| *Todiramphus* | *sanctus* | *sanctus* | UWBM | Bu68062 | PNG: Bismarck Archipelago; Schumann Is. (north coast New Britain Is.) |
| *Todiramphus* | *sanctus* | *sanctus* | UWBM | Bu72545 | AUSTRALIA: Queensland |
| *Todiramphus* | *sanctus* | *sanctus*^B^ | UWBM | Bu76296 | SOLOMON ISLANDS: New Georgia Is. |
| *Todiramphus* | *sanctus* | *sanctus* | AMNH | DOT12594 | INDONESIA: Sulawesi Is. |
| *Todiramphus* | *sanctus* | *vagans*^B^ | KUNHM | 14877 | NEW ZEALAND: Aukland; Warkworth |
| *Todiramphus* | *sanctus* | *vagans*^B^ | KUNHM | 14879 | NEW ZEALAND: Auckland; Waiheke Is. |
| *Todiramphus* | *saurophagus* | *saurophagus*^B^ | KUNHM | 27804 | PNG: Bismarck Archipelago; Nusalaman Is. |
| *Todiramphus* | *saurophagus* | *saurophagus*^B^ | UWBM | Bu60204 | SOLOMON ISLANDS: Kiaba Is. (north coast Isabel Is.) |
| *Todiramphus* | *saurophagus* | *saurophagus* | UWBM | Bu60326 | SOLOMON ISLANDS: Hekelake Is. (north coast Isabel Is.) |
| *Todiramphus* | *saurophagus* | *saurophagus* | UWBM | Bu69666 | SOLOMON ISLANDS: Hekelake Is. (north coast Isabel Is.) |
| *Todiramphus* | *tutus* | *atiu*^B^ | UWBM | Bu42503 | COOK ISLANDS: Atiu Is. |
| *Todiramphus* | *tutus* | *atiu* | UWBM | Bu42504 | COOK ISLANDS: Atiu Is. |
| *Todiramphus* | *tutus* | *mauke*^B^ | UWBM | Bu42603 | COOK ISLANDS: Mauke Is. |
| *Todiramphus* | *tutus* | *mauke* | UWBM | Bu42604 | COOK ISLANDS: Mauke Is. |
| *Todiramphus* | *tutus* | *tutus* ^*B^ | MHNG | HH7-60 | FRENCH POLYNESIA: Society Islands; Ra‘iatea Is. |
| *Todiramphus* | *tutus* | *tutus* ^*^ | MHNG | HH7-62 | FRENCH POLYNESIA: Society Islands; Ra‘iatea Is. |
| *Todiramphus* | *veneratus* | *veneratus* ^*B^ | MHNG | PO2-88 | FRENCH POLYNESIA: Society Islands; Tahiti Is. |
| *Todiramphus* | *veneratus* | *youngi* ^*B^ | MHNG | HH7-75 | FRENCH POLYNESIA: Society Islands; Mo‘orea Is. |
| *Todiramphus* | *veneratus* | *youngi* ^*B^ | MHNG | HH7-77 | FRENCH POLYNESIA: Society Islands; Mo‘orea Is. |
| Outgroup |  |  |  |  |  |
| *Actenoides* | *hombroni*^B^ |  | KUNHM | 19212 | PHILIPPINES: Mindanao Is. |
| *Syma* | *megarhyncha*^B^ |  | KUNHM | 7143 | PNG: Morobe Province |
| *Syma* | *torotoro*^B^ |  | KUNHM | 5215 | PNG |
| *Todiramphus* | *leucopygius*^B^ |  | KUNHM | 15882 | SOLOMON ISLANDS: Guadalcanal Is. |
| *Todiramphus* | *leucopygius* |  | KUNHM | 15901 | SOLOMON ISLANDS: Guadalcanal Is. |
| *Todiramphus* | *leucopygius* |  | KUNHM | 15902 | SOLOMON ISLANDS: Guadalcanal Is. |
| *Todiramphus* | *leucopygius*^B^ |  | AMNH | DOT6654 | SOLOMON ISLANDS: Isabel Is. |
| *Todiramphus* | *macleayii*^B^ |  | ANWC | Au33585 | AUSTRALIA |
| *Todiramphus* | *nigrocyaneus*^B^ |  | KUNHM | 5294 | PNG: Gulf Province |
| *Todiramphus* | *pyrrhopygius*^B^ |  | ANWC | Au32904 | AUSTRALIA |
| *Todiramphus* | *winchelli* | *nesydrionetes*^B^ | KUNHM | 14453 | PHILIPPINES: Tablas Is. |
| *Todiramphus* | *winchelli* | *nesydrionetes* | KUNHM | 14490 | PHILIPPINES: Tablas Is. |
| *Todiramphus* | *winchelli* | *nesydrionetes*^B^ | FMNH | F358323 | PHILIPPINES: Sibuyan Is. |
| *Todiramphus* | *winchelli* | *nigroroum*^B^ | KUNHM | 14302 | PHILIPPINES: Leyte Is. |
| *Todiramphus* | *winchelli* | *nigroroum*^B^ | KUNHM | 28186 | PHILIPPINES: Bohol Is. |

^†^ Samples from museum toepads.

^*^ Samples from unvouchered blood.

^B^ Samples included in BEAST divergence time analyses.

Table ESM2 (Electronic Supplementary Material).

| locus | primer name | 5’ to 3’ sequence |
| --- | --- | --- |
| CCDC132 | CCDC132H.Todi | CTCCAACTTGCATCAGCCTG |
|  | CCDC132L.Todi | CTGTCTAACTTCAAATACGACGAC |
|  | CCDC132H.Todi.int | GAGACCTCATTAGGCAGG |
|  | CCDC132L.Todi.int | AGTGCCGGTCTCTCTTTCTT |
| HMGB2 | HMG2H.Todi | GCTCTTGGCACGATATGCCG |
|  | HMG2L.Todi | GGTCTGAACAGTCGGCAAAAG |
|  | HMG2H.Todi.int | GGGATTTCCATGCTTACAGC |
|  | HMG2L.Todi.int | AGTGTTTGTCAGCCTTTTCCA |
| MUSK | MUSK.Todi.IntF | GTCCAGATGCTGCTGAATG |
|  | MUSK.Todi.IntR | TGACACACTCACTCATCCCTGT |
| ND2 | Todi190L | AATTAAATACTTCCTGGTCCAAG |
|  | Todi410L | ATCAACAATAATAAAATTTCC |
|  | Todi452L | AACATCTCACTCCCTAAACCC |
|  | Todi625L | ACCCTATTAACTTTCTACCTGTAC |
|  | Todi822L | CAAGAACTAACTAAACAAGA |
|  | Todi897L | ACCTACGTCTCGCATACTAC |
|  | Todi230H | GTCCTGTCTGYCAGGCAT |
|  | Todi232H | CTCATTGTCCTGTCTGTCAGGC |
|  | Todi465H | TGCTGATATTAAGGCTATTAGG |
|  | Todi618H | CGGTTATTAGGGAGTACAGG |
|  | Todi648H | ATTTTGTTGTGTTAAGTGAGAGG |
|  | Todi890H | GGTGATTGTTGAGTAGTATG |
| ND3 | 160L.ND3.Todi | AATCCGATTCTTCCTCAGTAG |
|  | 218L.ND3.Todi | GACCTAGAAATCGCCCTCC |
|  | 227H.ND3.Todi | TAGTTGGATGGCTCAGGGGAG |
| TGFβ2 | TGF5.Todi.int | CTCTGGGATGATTACCAGACCC |
|  | TGF6.Todi.int | CTCTCTGAGTAGGTGAGCACAT |

Table ESM3 (Electronic Supplementary Material).

| locus | aligned  length | category, chromosome # | substitution model | A, C, G, T frequency | variable sites | informative sites | source |
| --- | --- | --- | --- | --- | --- | --- | --- |
| CCDC132 | 730 | intron, 2 | HKY+I+G | 0.260, 0.159, 0.237, 0.344 | 66 | 39 | [51] |
| HMGB2 | 533 | intron, 4 | HKY+I+G | 0.25, 0.25, 0.25, 0.25 | 64 | 56 | [51] |
| MUSK | 600 | intron, Z | HKY+G | 0.284, 0.198, 0.210, 0.309 | 55 | 37 | [52] |
| TGFβ2 | 552 | intron, 3 | HKY+I | 0.25, 0.25, 0.25, 0.25 | 43 | 28 | [53] |
| ND2+ND3 | 1041+351 | mitochondrial |  |  |  |  |  |
|  |  | codon pos. 1: | HKY+G | 0.351, 0.315, 0.152, 0.182 | 108 | 89 | [54] |
|  |  | codon pos. 2: | HKY+I | 0.181, 0.332, 0.115, 0.372 | 48 | 31 |  |
|  |  | codon pos. 3: | GTR+I+G | 0.467, 0.360, 0.069, 0.104 | 271 | 219 |  |
